# On the Course of the Amyloid Degeneration

**Published:** 1856-06

**Authors:** Rudolph Virchow


### RECORD OF MEDICAL SCIENCE.

*On the Course of the Amyloid Degeneration.*—By Rudolph Virchow. (Abstracted from the Archiv. f. Patholog. Anatomie und Physiologie. Bd. viii., p. 364.)

In former communications on the subject of "amyloid degeneration" the Author was able to adduce, as instances of the affection, besides the *corpora amlyacea* in the nervous system, only the waxy degeneration of the spleen, liver, and kidneys; but since then some more recent cases have afforded him the opportunity of extending his researches, and of making, as he thinks, a very important advance in the knowledge of the remarkable changes included under the term.

In all these cases there existed chronic, and very considerable disease in some part of the *osseous system*. Even in his former communication, respecting the "waxy spleen," he had noticed that it was especially in persons affected with chronic disease of the bones that this form of degeneration of the organ was presented, and he has since seen scarcely a single case in which the same complication did not exist. This frequent association cannot, he thinks, be explained except upon the supposition that the disease in the bone exerts a determinate influence upon the production of the affection in the spleen, liver, and kidneys. It is usually the case that primary, long-continued disease of the bones, especially *caries* and *necrosis* of the larger bones or portions of the skeleton, in their subsequent course, induce cachexia and dropsy, and particularly *albuminuria* and degeneration of the kidneys, but how is the connexion between the primary and secondary affections to be explained? Two hypotheses, with respect to this, might be entertained, either the disease in the osseous system may so far interfere with the general nutrition that the constituent elements of the spleen, kidneys, and liver may be deprived of their normal supply of nutriment, and disposed to undergo the amyloid change, or the disease in the bones may actually produce the amyloid matter, which is deposited secondarily in the other organs. In the former case there would be a peculiar metamorphosis, an idiopathic, morbid change in the elements of the spleen, liver, and kidneys; and in the second an instance of metastasis, in which the glandular organs would be merely the seat of the deposition of the morbid material.

Hitherto Virchow has not found in the *bone* itself a substance corresponding to that which occurs in the abdominal glands, whilst he has always detected its presence in the *cartilages.* In an aged individual, who presented in many of the joints the changes peculiar to senile arthritis, the pubic symphysis in particular, towards the interior aspect, was much enlarged, and unusually moveable. When cut across, there was apparent in the middle of it an irregular, vertical fissure, with uneven, somewhat tuberous walls, and without any fluid contents. The layers of cartilage on each side were considerably thickened, of a dirty, yellowish color, and very unequal density; the parts immediately contiguous to the fissure were more especially softened in places, greasy, and as it were, broken up, so that portions, of considerable size, were almost separated from the rest, or were held together only by slender connexions. Microscopic examination disclosed a great variety of constituents. The cartilage cells were generally enlarged, their capsules very thick and wide; in many places considerable-sized groups of them might be observed in a proliferous state, but in some might also be seen minute, roundish, or flattened corpuscles. Towards the surface of the fissure many cartilage cells were in a state of fatty degeneration, the matrix being, at the same time, transformed into a soft, clouded, streaked, and granular substance, in which the presence of cholesterin was here and there perceptible. In these situations the condition might be described as "*atheromatous degeneration*," similar to that which takes place in the arteries. Crystalline cholesterin existed only on the surface, beneath which, however, the matrix presented numerous alterations; isolated portions were composed, in great part, of the unchanged, hyaline, dense substance, close to which might be noticed considerable tracts and masses in which the matrix was streaky and fibrous. The fibres in some parts resembled the rigid filaments in the well-known asbestos-like portions of the costal cartilages, and in others assumed more the aspect of hard, wavy, and strongly refractive *striæ.* On the addition of solutions of iodine, either the simply aqueous, or made with iodide of potassium, some portions of the microscopic section at once assumed an intense reddish-yellow (iodine-red) color, whilst others remained perfectly clear and colorless; the greater part presented a yellowish, and, on more prolonged action of the reagent, a yellowish-brown hue. If sulphuric acid or chloride of zinc be now added, the reddish-yellow spots are immediately rendered of a violet, or occasionally, bright blue color, although a strong reddish tinge is always retained. Under the action of a very concentrated solution of iodine, also the color becomes at once dark red, or nearly violet-red, especially when the section so treated is dried and again moistened with water. The places in the section where the iodine reaction took place, might be very distinctly recognized, even by the naked eye, as dark, reddish, or blackish-red points, particularly when thin sections were viewed over a clean, white surface. When examined with the microscope it was readily seen that it was not cholesterin in any form which afforded the simple reaction with iodine; as is usual, this substance, even after the addition of iodine, remained colorless, and did not exhibit any of the often-noticed changes of color, except under the energetic action of sulphuric acid or of chloride of zinc.

It was now a point of much interest to determine in which of the structural elements the reaction took place; with respect to which it was at once evident that both the matrix-substance and the corpuscles participated in it, either each singly, or both, though less extensively, conjointly. Of the corpuscles, again, it was quite evident that it was the thick capsules which afforded the deepest coloring, which was intense in proportion as the corpuscles were of larger size, and more free in the surrounding matrix; but in some places the true cell (contents of the capsules) also appeared to be similarly affected; and especially in the smaller ones, Virchow often noticed the entire corpuscles colored red or violet throughout.

It was remarkable that no microscopic characters could be discerned, from which it might be concluded *a priori* whether the parts would be acted upon by the iodine or not; neither in the matrix, nor in corpuscles, did the spots, which were afterwards colored, exhibit before the addition of the iodine, any difference from those which remained uncolored; nor, excepting the rather remarkable *microscopical* condition of the whole cartilage, could it be said that these cartilages presented any appearance by which they could be distinguished from many other senile cartilages in which the reaction did not occur. This circumstance, with regard to the cartilages, is perhaps of the more importance to be noted, as a strong contrast in this respect was presented in other parts, and especially in the glandular organs, in all of which, especially in more advanced stages of the affection, in the portions where the amyloid change had taken place, a degree of softening independent of any reagent might be recognised, and particularly the presence of a brilliant, pale, thickening substance."

Two additional cases are given, one of a boy who had died of albuminuria and dropsy, following *spondylarthrocasis*, the other of a man who had necrosis of the *femur*; in the first the degeneration was very extensive, and implicated not only all the lymphatic glands, but was also manifested in the vessels of the gastric mucous membrane, in those of the mucous membrane of the œsophagus, and of the whole intestinal canal, particularly in the small intestine. "It was limited in all parts to the fine arterial vessels of the mucous membrane, or at most involved only those of the uppermost layer of the sub-mucous tissue, and it might be traced to some distance into the arterial side of the capillaries." On the application of iodine, a very deep dark violet color was manifested. The mucous membrane in all these parts had a very pale aspect. It was slightly thickened, unusually transparent, and in parts of gelatinous consistent.

With respect to the course followed by the morbid change, it appears indubitable that the incitement to it proceeds from the diseased bones, whence it extends progressively to the lymphatic glands, then to the spleen, and ultimately to several of the secretory organs. Among these, the first to suffer are invariably the kidneys, then the liver, probably lastly the mucous membrane of the digestive organs ; and it is a circumstance of the greatest interest, that both in the kidneys and in the digestive mucous membrane the morbid change always commences in the secretory vessels, in the same way as in the lymphatic glands, the spleen, and renal *papillæ,* the vessels, and especially the arterial, are very early affected. In all cases the normal tissue is removed in proportion to the amount of the new deposit, and it is not the individual elements which degenerate each separately, but the change involves all equally, so that the ultimate products present a very uniform, homogeneous constitution. From all that appears therefore, it is highly probable that the affection consists rather in a metastasis of a material formed in the site of the original diseased action, that is to say, in the bones, and which is transported to the different parts in a state of solution.

The constitution of the deposit is not everywhere alike, as has been before remarked by Virchow ('Archiv. Bd.' iii. p. 144) and Meckel. In particular, it would seem, that the substance in cases of less complete deposition, though assuming a beautiful red color even under iodine alone, receives only an indistinct violet tint on the addition of sulphuric acid, and is never rendered blue. This was the case very remarkably in a boy, fourteen years of age, affected with disease of the lumbar vertebræ, whose liver weighed 5 lbs. 13 oz., the spleen about 7½ oz., one kidney nearly 4 and the other 3½ oz. ; in whom the entire parenchyma of the liver, the spleen in its pulp, the kidneys in the *glomeruli,* the afferent arteries, and in the *papillæ,* exhibited the most complete waxy degeneration. With sulphuric acid the iodine-red color was deepened, but rapidly became of a dirty violet, or rather of a dark bluish-red hue, and in parts greenish. In this case, therefore, the substance existed either in a less perfect form, or was mixed with other matters.

*Quarterly Journal of Microscopical Science.*

